# Clinicopathological and Prognostic Significance of Cancer Antigen 15-3 and Carcinoembryonic Antigen in Breast Cancer: A Meta-Analysis including 12,993 Patients

**DOI:** 10.1155/2018/9863092

**Published:** 2018-05-02

**Authors:** Xuan Li, Danian Dai, Bo Chen, Hailin Tang, Xiaoming Xie, Weidong Wei

**Affiliations:** Department of Breast Oncology, Sun Yat-sen University Cancer Center, State Key Laboratory of Oncology in South China, Collaborative Innovation Center for Cancer Medicine, 651 East Dongfeng Road, Guangzhou, 510060, China

## Abstract

**Purpose:**

The prognostic role of serum cancer antigen 15-3 (CA15-3) and carcinoembryonic antigen (CEA) in breast cancer remains controversial. In this study, we conducted a meta-analysis to investigate the prognostic value of these two markers in breast cancer patients.

**Methods:**

After electronic databases were searched, 36 studies (31 including information regarding CA15-3 and 23 including information regarding CEA) with 12,993 subjects were included. Based on the data directly or indirectly from the available studies, the hazard ratios (HRs) and odds ratios (ORs) and their 95% confidence intervals (CIs) were pooled according to higher or lower marker levels.

**Results:**

Elevated CA15-3 or CEA was statistically significant with poorer DFS and OS in breast cancer (multivariate analysis of OS: HR = 2.03, 95% CI 1.76–2.33 for CA15-3; HR = 1.79, 95% CI 1.46–2.20 for CEA; multivariate analysis of DFS: HR = 1.56, 95% CI 1.06–1.55 for CA15-3; HR = 1.77, 95% CI 1.53–2.04 for CEA). Subgroup analysis showed that CA15-3 or CEA had significant predictive values in primary or metastasis types and different cut-offs and included sample sizes and even the study publication year. Furthermore, elevated CA15-3 was associated with advanced histological grade and younger age, while elevated CEA was related to the non-triple-negative tumor type and older age. These two elevated markers were all associated with a higher tumor burden.

**Conclusions:**

This meta-analysis showed that elevated serum CA15-3 or CEA was associated with poor DFS and OS in patients with breast cancer, and they should be tested anytime if possible.

## 1. Introduction

Breast cancer has the highest incidence rate and second highest mortality rate among female cancers [1]; thus, its survival prognosis concerns doctors and patients. Prognostic factors are those clinicopathological parameters that are associated with tumor outcomes. In breast cancer, the most used prognostic factors include patient characteristics (age and menstrual status), tumor features (tumor size, node status, and TNM stage), tumor tissue markers (estrogen receptor, progesterone receptor, HER-2 status, and ki-67 status), and genetic markers (BRCA1/2) [2–8]. Using prognostic factors alone or combined to predict a worse outcome of patients and take advanced treatment early may improve patient survival. Identifying more available and convenient factors is very important.

Serum markers can be easily achieved, and they play an important role in many malignant tumors [9–11], but their role in breast cancer remains controversial. There is some correlation between tumor markers and tumor clinicopathology [12], and when the acquisition of tissue specimens is not available, in some cases, these markers may offer useful information about the phenotype of the breast cancer at an early stage [13]. Serum tumor markers in breast cancer include cancer antigen 15-3 (CA15-3), cancer antigen 27.29 (CA27.29), carcinoembryonic antigen (CEA), tissue polypeptide antigen (TPA), circulating extracellular domain of HER-2, and tissue polypeptide-specific antigen (TPS) [14, 15]. Among these, CA15-3 and CEA are the most used and recommended markers. The prognostic value of CA15-3 had been approved by some studies [16–18], while other studies reported negative results [19–21]. Ebeling et al. in a study of 1046 patients reported CA15-3 in univariate but not in multivariate analysis to be a predictor of a worse outcome [22]. In a review article, Duffy collected at least 10 studies and descriptively indicated that higher CA15-3 may be associated with a poor outcome but did not perform pooled analysis to confirm the results [14]. CEA is less widely investigated as a prognostic factor than CA15-3 because it has a less positive rate and is more controversial. Some studies have reported that CEA is not a predictor in primary and metastasis breast cancer [20, 21, 23, 24], but others have reported that high concentrations of CEA were related to a poor prognosis in breast cancer [17, 25, 26]. These above contradictory results of CA15-3 and CEA in breast cancer regarding their prognostic value may be due to small sample sizes, variable study designs, or other biases in each single study. We searched and combined available studies in this meta-analysis to explore the prognostic role of CA15-3 and CEA in breast cancer as well as their relationship with tumor clinicopathological factors, hoping to help medical workers affirm and properly use these two serum markers.

## 2. Materials and Methods

### 2.1. Search Strategy

We performed a systematic literature search using PubMed, Embase, and Web of Science. The search strategy terms are as follows: “CA15-3” (e.g., “cancer antigen 15-3,” “carbohydrate antigen 15-3,” and “cancer associated antigen 15-3”), “CEA” (e.g., “carcinoembryonic antigen,” “carcino-embryonic antigen,” and “carcino embryonic antigen”), “prognosis” (e.g., “outcome,” “survival,” “prognostic,” “mortality,” and “recurrence”), and “breast cancer” (e.g., “breast neoplasms” and “breast carcinoma”). The search was updated to July 15, 2017. The relevant articles were also manually checked.

### 2.2. Inclusion and Exclusion Criteria

The inclusion criteria for this meta-analysis were as follows: (i) the diagnosis of breast cancer was defined based on pathological results, (ii) CEA and CA15-3 were derived from a serum test, (iii) the correlation of serum CEA and/or CA15-3 with overall survival (OS) and/or disease-free survival (DSS) and/or disease-free survival (DFS) and/or progression-free survival (PFS) was reported, and (iv) hazard ratios (HRs) and 95% confidence intervals (CIs) can be directly or indirectly received. The exclusion criteria were as follows: (i) abstracts, letters, reviews, case reports, proficient opinions; (ii) literature not written in English; (iii) studies without hazard ratios (HRs) and 95% CIs; (iv) studies with duplicate data with few cases; and (v) nonhuman studies.

### 2.3. Data Extraction

We extracted data by two independent authors and through discussion or consensus with a third author regarding controversial data. The following items were recorded from each study: name of the first author, publication year, country, data inclusion criteria of each study, overall sample size, mean age and follow-up time of patients, tumor data of the TNM stage, time of serum sample collection, cut-off values of CEA and/or CA15-3 and the associated clinicopathological factors, and survival data, including HRs with 95% CIs. We used the Newcastle–Ottawa Scale (NOS) to assess the quality of each study by two authors. The scores included three parts: the selectivity of patients (0–4), comparability of groups (0–2), and assessment of outcome (0–3). Studies with scores > 5 were considered high quality.

### 2.4. Statistical Analysis

We directly obtained all multivariate data and some univariate data of HRs and 95% CIs from the literature. Few univariate data were estimated according to the methods illustrated by Parmar et al. and Tierney et al. using Engauge 4.0 and an HR calculation spreadsheet [27, 28]. HR > 1 indicated a worse prognosis in breast cancer patients. All the studies with HRs and 95% CI were classified into three groups (studies with primary TNMs I–III to group 1; studies with metastatic breast cancer to group 2; and studies with primary TNMs I–IV or unclear stage into group 3), and the pooled results included all the three study groups. The relationship between CEA and/or CA15-3 with clinicopathological parameters was determined using ORs and 95% CIs.

In this meta-analysis, Cochran's *Q* test was undertaken to assess the heterogeneity. All the studies first used fixed-effect (Mantel–Haenszel method) models to calculate the pooled results, but if (heterogeneity *p* value) Ph < 0.1 or *I* ^2^ > 50%, which suggested significant heterogeneity, we changed to a more conservative random-effect (DerSimonian–Laird method) model to confirm the results. Subgroup analysis was conducted to explore and explain the heterogeneity. Publication bias was first assessed by Begg's funnel plot, and the result of pr > |*z*| > 0.05 was regarded as no publication bias. If bias was not certain, Egger's bias indicator test was used to reconfirm. Results with *p* < 0.05 were considered statistically significant, and all the results were two sided.

## 3. Results

### 3.1. Study Characteristics

**Figure 1:**
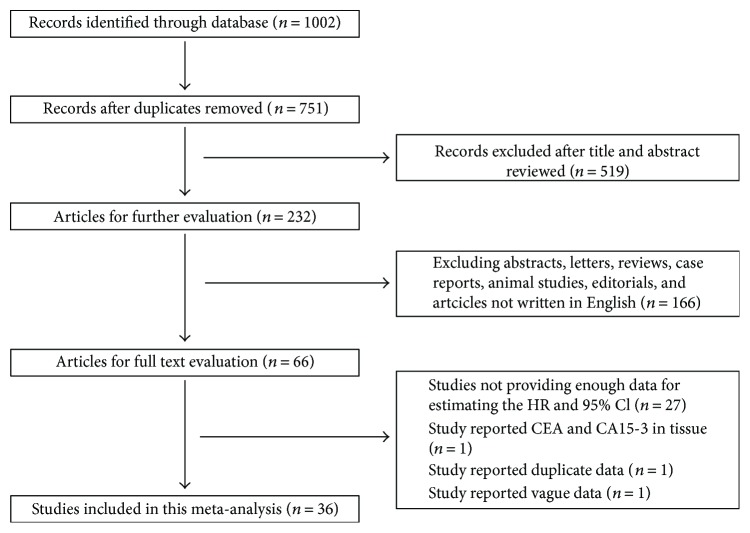
Flow chart of the included studies.

The initial search information retrieved 751 articles. After careful inspection, 36 studies published between 1986 and 2017 that comprised 12,993 breast cancer patients met our inclusion criteria and were finally included in this meta-analysis [3, 13, 16–21, 23–26, 29–52]. The specific processes of the literature selection are shown in Figure 1. Among them, 18 studies were from Europe and the United States, 14 studies were from Asia, and 4 studies were from Africa. Twenty-eight studies concerned the initial treatment of breast cancer patients, and eight were focused on metastatic breast cancer patients. Twenty-four studies collected serum specimens before treatment, and twelve studies collected serum specimens after surgery or at the time of recurrence and metastases. Thirty-one studies provided the survival information of serum CA15-3, and its cut-off values ranged from 20.11 to 77 U/ml; meanwhile, twenty-three studies provide the survival information of serum CEA, and its cut-off values ranged from 2.5 to 20 ng/ml. Clinicopathology information correlated with serum CA15-3 or CEA could be extracted from sixteen studies. Further information of articles in this study is shown in Table 1.

### 3.2. CA15-3 and DFS in Breast Cancer

**Figure 2:**
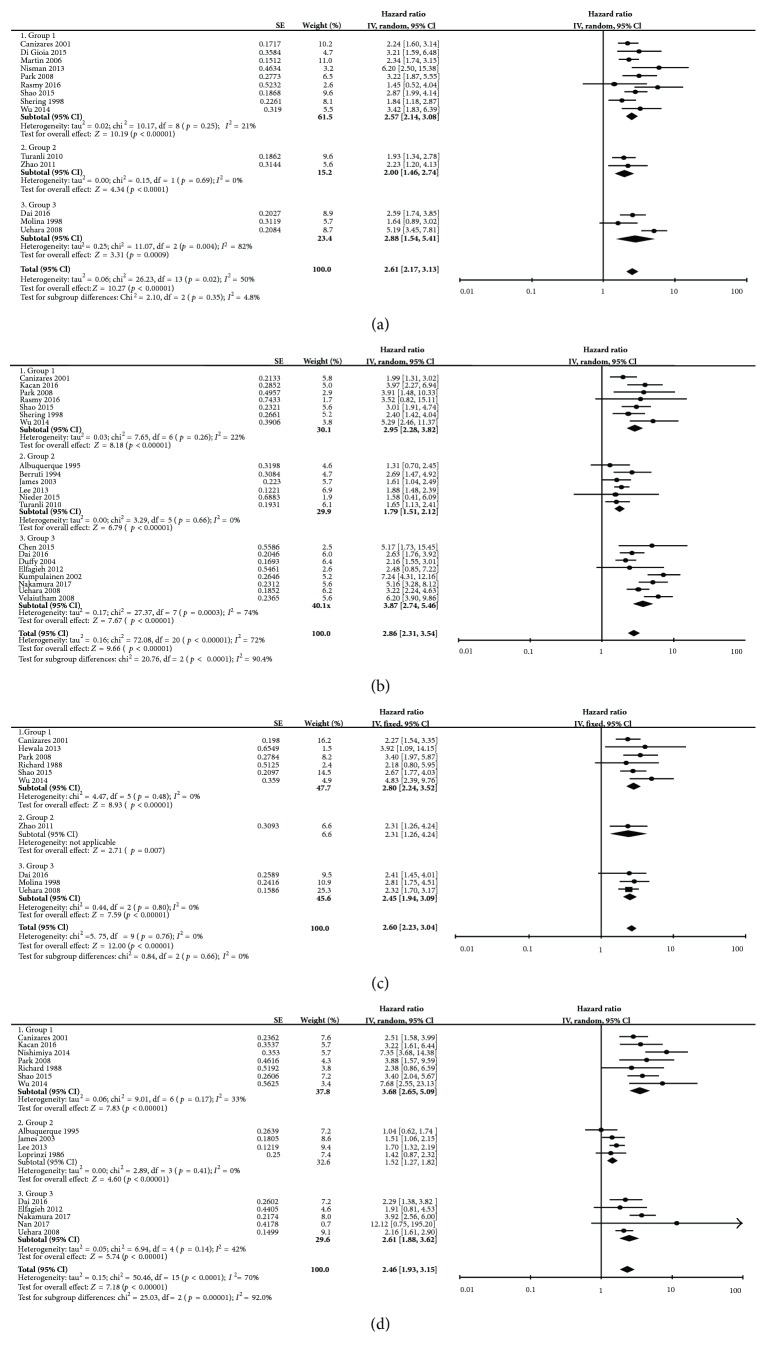
Meta-analysis of the univariate result associations between CA15-3/CEA and OS/DFS of breast cancer. The results are presented as an individual and pooled HR and 95% CI: (a) DFS of CA15-3; (b) OS of CA15-3; (c) DFS of CEA; (d) OS of CEA.

**Figure 3:**
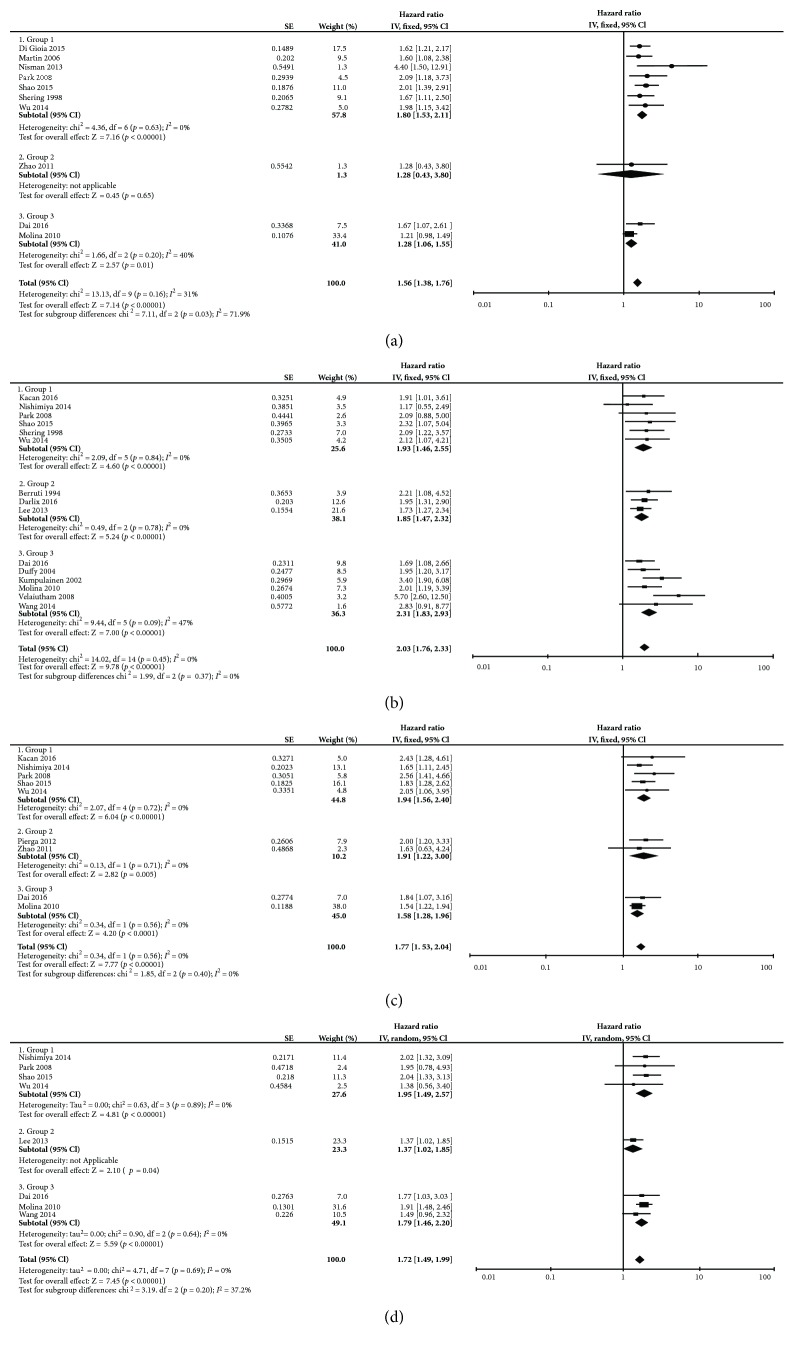
Meta-analysis of the multivariate result associations between CA15-3/CEA and OS/DFS of breast cancer. The results are presented as an individual and pooled HR and 95% CI: (a) DFS of CA15-3; (b) OS of CA15-3; (c) DFS of CEA; (d) OS of CEA.

Fourteen studies provided univariate HRs and 95% CIs of DFS according to the cut-off value of serum CA15-3 (Figure 2(a)). Combined data from the fourteen studies showed that higher CA15-3 was significantly corrected with poor DFS with a pooled univariate HR of 2.61 (95% CI: 2.17–3.13) with significant heterogeneity (*I* ^2^ = 50%, Ph = 0.02, random effects model). Ten studies provided multivariate HR and 95% CI information of DFS concerning CA15-3 (HR = 1.56, 95% CI: 1.38–1.76, *I* ^2^ = 31%, Ph = 0.16, fixed-effect model; Figure 3(a)). Only one study was included in group 2, and it showed that CA15-3 was not an independent prediction factor for DFS in metastatic breast cancer (group 2: HR = 1.28; 95% CI 0.43–3.80; *p* = 0.65). Among the fourteen studies, two studies reported the results of PFS that was the result of group 2, which showed that HR was 2.00 (95% CI: 1.46–2.74) for PFS without significant heterogeneity (*I* ^2^ = 0%, Ph = 0.69). In multivariate subgroup analysis, the results in European countries and the United States, compared with those in Asian and African countries, showed significant subgroup heterogeneity and lower cut-off values in pretreatment patients had higher prognostic values (Table 2).

### 3.3. CA15-3 and OS in Breast Cancer

Twenty-one studies had extractable univariate OS HR data concerning CA15-3 (Figure 2(b)). Elevated CA15-3 indicated a shorter OS (HR = 2.86, 95% CI: 2.31–3.54, *p* < 0.001) with obvious heterogeneity (*I* ^2^ = 72%, Ph < 0.001, random-effect model). Heterogeneity was significantly different among the three groups as the pooled HR was the lowest in group 2 (group 1: HR = 2.95, 95% CI 2.28–3.82, *I* ^2^ = 22%, Ph = 0.26; group 2: HR = 1.79, 95% CI 1.51–2.12, *I* ^2^ = 0%, Ph = 0.66; group 3: HR = 3.87, 95% CI 2.74–5.46, *I* ^2^ = 74%, Ph = 0.0003). Stratification by ethnicity found that CA15-3 was a negative predictor for OS in both European countries and the United States (HR = 2.35, 95% CI: 1.74–3.18, *p* < 0.001) and in Asian and African countries (HR = 3.50, 95% CI: 2.59–4.71, *p* < 0.001). Fifteen studies reported multivariate data of OS (Figure 3(b)). Elevated CA15-3 was an independent prognosis predictor for OS in breast cancer with a merged HR of 2.03 (95% CI: 1.76–2.33, *p* < 0.001), and heterogeneity was not significant (*I* ^2^ = 0%, Ph = 0.45). Among these three groups, no significant heterogeneity was seen. The subgroup analysis results are shown in Table 2.

### 3.4. CEA and DFS in Breast Cancer

The univariate HR of ten studies and multivariate HR of nine studies of CEA and DFS could be obtained. Pooled univariate data showed that high CEA was associated with worse DFS with HR = 2.60 (95% CI: 2.23–3.04; no significant heterogeneity *I* ^2^ = 0%, Ph = 0.91, fixed-effect model; Figure 2(c)). The meta-analysis of multivariate data indicated that CEA was an independent predictor for DFS with a HR of 1.77 and the 95% CI ranging from 1.53 to 2.04 (no significant heterogeneity *I* ^2^ = 0%, Ph = 0.82, fixed-effect model; Figure 3(c)). There was no heterogeneity among the three groups in either univariate or multivariate analysis.

### 3.5. CEA and OS in Breast Cancer

Meta-analysis of sixteen univariate HR results showed that breast cancer patients with higher CEA levels were significantly associated with shorter OS (HR = 2.46, 95% CI: 1.93–3.15, *p* < 0.001; heterogeneity: *I* ^2^ = 70%, Ph < 0.001; random-effect model; Figure 2(d)). Classification according to the three groups can partly explain the heterogeneity of the pooled results as each group showed no significant heterogeneity but subgroup heterogeneity differences were significant (group 1: HR = 3.68, 95% CI 2.65–5.09, *I* ^2^ = 33%, Ph = 0.17; group 2: HR = 1.52, 95% CI 1.27–1.82, *I* ^2^ = 0%, Ph = 0.41; group 3: HR = 2.61, 95% CI 1.88–3.62, *I* ^2^ = 42%, Ph = 0.14). Pooled multivariate HR results also provided similar results as higher CEA revealed an HR of 1.72 (95% CI: 1.49–1.99) in OS without significant heterogeneity (Figure 3(d)). We performed subgroup analysis and found that, in univariate analysis, CEA derived from primary breast cancer had a higher HR than that from metastatic breast cancer. Subgroup analysis was also performed according to different cut-off values, ethnicity, and publication year (Table 2).

### 3.6. CA15-3 and CEA with Tumor Clinicopathological Parameters

We extracted twelve factor data according to the cut-off of the high and low groups of CA15-3 or CEA (Table 3). The results demonstrated that patients aged ≤35 years had a higher rate of CA15-3 increase (OR = 1.83, 95% CI: 1.20–2.80, *p* = 0.05), while younger ages had a lower rate of CEA rise (OR = 0.52, 95% CI: 0.29–0.94, *p* = 0.03). Higher rate of CA15-3 and CEA rise were correlated with tumor burden as larger tumor size, lymph node metastasis, and advanced TNM stages. Higher CA15-3 rates were also related to an advanced tumor histological grade (OR = 0.63, 95% CI: 0.46–0.87, *p* = 0.005). However, non-triple-negative-type breast cancer was more likely to associated with a CEA level increase (OR = 2.08, 95% CI: 1.30–3.33, *p* = 0.003).

### 3.7. Publication Bias

Begg's funnel plot was used to evaluate publication bias. All the results except one showed no significant publication bias as the values of pr > |*z*| > 0.05. The multivariate pooled results of OS in CA15-3 had pr > |*z*| = 0.01 for Begg's test. Next, we carefully examined Begg's funnel plot and performed Egger's test for this result. Egger's test showed *p* > |*t*| = 0.104, and Begg's funnel plot was approximately symmetrical; thus, we believe that publication bias did not show a significant difference (Supplement Figure 1).

## 4. Discussion

The prognostic factors of breast cancer include tumor biopathological factors such as tumor burden, hormone receptors, HER-2, and Ki-67 levels [53, 54]. All these factors should be determined directly from tumor tissue through biopsy or surgery. It would be desirable if serum markers were used as available factors for prognosis. At present, the use of serum tumor markers in breast cancer is less well established because of its lower sensitivity and specification. Many studies have reported a low positive rate of CA15-3 and an even lower rate of CEA [17, 34]. Without more powerful serum markers, although imperfect, CA15-3 and CEA remain the most commonly used biomarkers in breast cancer and are recommended for practical use by the American Society of Clinical Oncology (ASCO). However, because of insufficient data, the use of CA15-3 and CEA as screening, diagnostic, and staging tests to detect recurrence and monitoring the response to treatment alone is not recommended by ASCO as well as its prognostic functions [15]. However, the European Group on Tumor Markers has recommended the use of CEA and CA15-3 to assess the prognosis in breast cancer [55]. Our meta-analysis was focused on serum CA15-3 and CEA prognostic ability and their best use of these two markers in breast cancer.

The meta-analysis indicated that an elevated CA15-3 level significantly corresponded with poor DFS and OS of breast cancer. In our analysis, serum CA15-3 had prognostic ability in the pretreatment of primary early-stage and primary all-stage breast cancer patients. It also showed prognostic values in metastasis in OS but not in multivariate analysis of DFS because of only one study reported this result, and it may show great bias. The reason why CA15-3 can predict the prognosis in breast cancer is not fully clear, but as CA15-3 is the soluble form of MUC1, this may be related with the function of MUC1. MUC1 was reported to not only allow the cancer cells to escape the immune system but also promote cancer cell migration by activating some membrane receptors [56–59]. Serum CA15-3 in metastasis studies had lower HR than that in primary studies. The cause may be that CA15-3 is closely corrected with tumor burden, and in pretreated tumors, it is consistently associated with tumor characteristics that can best predict the prognosis of survival [60]. Nieder et al. found that overall survival was significantly associated with CA15-3 in brain metastasis patients with breast cancer and this trend was not found in other cancers [32]. Subgroup analysis indicated that different cut-off values of pretreatment tumor studies had different predictive abilities, and the lower cut-off value studies had higher HRs in DFS and OS. However, Keshaviah et al. combined 7 study group trials and found a lower cut-off would decrease the predictive value of CA15-3 [61]. This different result may be because Keshaviah used serum CA15-3 tested at any time during disease; however, in the present study, we used the pretreatment serum sample only. We also found differences according to country. European countries and the United States had lower HRs than Asian and African countries because developed countries had improved screening methods and self-consciousness. As the tumor can be found at an early stage in developed countries [62–64], CA15-3, used as a predictor, may be more useful in developing countries, and this region-specific use of tumor marker test phenomenon was also found by Ramsey et al. [65]. Though CA15-3 seems to be able to assess the prognosis of breast cancer, there was lack of prospective testing to clarify it which limited its practical use in the clinical setting.

The CEA levels are less likely to be elevated than CA15-3; however, in symptomatic breast cancer patients, CEA sensitivity increased, and some studies found that CEA are able to correlate with the stage of disease as well as prognosis though these results are also in dispute. De Jong-Bakker et al. reported the pretreatment level of CEA had no relationship with all groups of patients regarding prognosis as the same results of other small-sample studies report [33, 34, 44, 66]. However, in our pooled results, higher serum CEA had lower DFS and OS both in univariate and multivariate analyses. Serum CEA showed no significant difference in HR in primary and metastasis breast cancer regarding DFS but had a higher HR of primary cancer than metastasis regarding OS. Subgroup analysis showed a lower cut-off value, and studies in Asian and African countries had higher HRs with the same tendency as CA15-3. In clinical use, CEA can be informative when levels of CA15-3 remain below the cut-off point, but for now, no high-level evidence study has verified it [67].

CA15-3 and CEA have been reported to be associated with clinicopathological parameters [68]. Both the CA15-3 and CEA levels were elevated with a larger tumor size, positive lymph node metastasis, and advanced TNM stage. A higher elevation of the markers does not indicate that their prognostic value has increased. In CA15-3 and CEA with tumor burden prognostic analysis, Molina et al. found that CEA had prognostic values in node-positive or node-negative breast cancer, but CA15-3 showed a prognostic value only in the node-negative patients [47]. Gion et al. also found that CA15-3 was significantly associated with the prognosis in node-positive cases not in node-negative cases [69]. In patients younger than 35 years and older, the results in CA15-3 and CEA had significant differences and were reversed. Young patients are more likely to have elevated CA15-3; however, older patients are more likely to have elevated CEA. This can inspire doctors to use different cut-off values in young and older breast cancer patients to best manage young patients. The molecular types of breast cancer may also be different in tumor markers expressed. Triple-negative breast cancer may more likely show high CEA according to a study that included 247 triple-negative breast cancer patients, in which the best cut-off value using the X-Tile program was 6 ng/ml higher than the recommended 5 ng/ml [25]. Li et al.'s study and other studies found that the CA15-3 levels showed a significant difference according to molecular subtype [60, 70], but the pooled results of our study saw no difference.

Several limitations need to be carefully considered in this analysis. First, almost all the included studies in this analysis were retrospective, making them more susceptible to some biases. Though we could not exclude all potential residual confounding, the asymmetry in the funnel plots at least showed the absence of publication bias, thus maintaining the substantial consistency of the results. Second, the publication year of the included studies in this analysis ranged from 1986 to 2017. This long period led to great differences; thus, we performed subgroup analysis and used the cut-off year of 2010 to limit the heterogeneity. Third, some studies have found that CA15-3 and/or CEA only had prognostic value according to molecular tumor types, but we could not identify sufficient studies to pool the results according to different molecular types of breast cancer. Fourth, although we performed multivariate analysis of CA15-3 and CEA as prognostic factors, the multivariate analyses were adjusted to different factors in different studies; thus, the pooled results using CA15-3 and/or CEA as an independent predictor should be carefully considered.

In conclusion, we found that both CA15-3 and CEA were good prognostic factors for poor DFS and OS when abnormal levels were found in breast cancer patients. Furthermore, higher CA15-3 or CEA had more significant prognostic significance for pretreatment primary breast cancer, a cancer occurring in Asian and African countries. Elevated CA15-3 and CEA were closely associated with age and tumor burden. In the future, more studies with uniform cut-off values, large-scale data, and good designs are needed to validate our conclusion.

## Figures and Tables

**Table 1:** Main characteristics of all the studies included in the meta-analysis.

Study	Year	Study region	Study continent	Tumor type	Sample size	Follow-up time mean (months)	Patients average age (years)	Serum sample collected time	Stage	CA15-3 cut-off value (U/ml)	Outcome	CEA cut-off value (ng/ml)	Outcome	Whether had association of clinicopathological characteristics with CA15-3 or CEA	NOS score
Albuquerque	1995	UK	Europe	Metastatic breast cancers	85	N	60.52	Pretreatment	M	40	OS	4	OS	No	6
Berruti	1994	Italy	Europe	Advanced breast cancer	115	35	56	At first recurrence of disease before the start of any endocrine or cytotoxic therapy	M	30	OS	N	N	No	5
Canizares	2001	Spain	Europe	Primary early breast cancer	364	72	56	Pretreatment	I, II, III	40	DFS, OS	6	DFS, OS	No	7
Chen	2015	China	Asia	Breast cancer with positive lymph nodes	220	36	49.0	Pretreatment	II, III, IV	28	OS	N	N	No	7
Dai	2016	China	Asia	Triple-negative breast cancer	247	120	46.8	Pretreatment	I–IV	21.8	DFS, OS	6.0	DFS, OS	Yes	7
Darlix	2016	France	Europe	Metastatic breast cancers	250	40.8	58.4	Not pretreatment	M	30	OS	N	N	No	6
Di Gioia	2015	Germany	Europe	Early-stage breast cancer	241	41.5	57	Pretreatment	I, II, III	24	DFS, CSS	N	N	Yes	7
Duffy	2004	UK	Europe	Breast cancer	600	75.24	N	Pretreatment	Not clear	30	OS	N	N	Yes	7
Elfagieh	2012	Libya	Africa	29 had locoregional breast cancer and 21 advanced breast cancer	50	28	N	Not clear	I–IV	35.57	OS	8.82	OS	Yes	6
Giovanella	2002	Italy	Europe	Primary breast cancer	212	26	53.6	Pretreatment	I–IV	30	OS	5	OS	No	7
Hewala	2013	Egypt	Africa	Stages II and III breast cancer	100	50	48.36	Pretreatment	II, III		N	4.88	DFS	No	8
James	2003	UK	Europe	Bone metastasis breast cancer	213	N	N	After metastasis	M	35	OS	10	OS	No	7
Kacan	2016	Turkey	Europe	Early-stage breast cancer	448	50	51	Pretreatment	I, II, III	31.3	DFS, OS	5	DFS, OS	No	8
Kumpulainen	2002	Finland	Europe	Primary breast cancer	272	124.8	54	In most patients, CA15-3 was assessed pre-first treatment; in the minority, by 4 weeks after the operation	0–IV	30	OS	N	N	Yes	7
Lee	2013	Korea	Asia	Metastatic breast cancers	349	18		At recurrence	M	20.11	OS	3.88	OS	Yes	6
Loprinzi	1986	South Africa	Africa	Metastatic breast cancers	97	N	N	After recurrence	M	N	N	5	OS	Yes	6
Martin	2006	Spain	Europe	Primary early breast cancer	818	43	59	Pretreatment	I, II, III	30	DFS	N	N	Yes	7
Molina	1998	Spain	Europe	Primary breast cancer	413	N	N	Pretreatment	I–IV	35	DFS	5.0	DFS	No	5
Molina	2010	Spain	Europe	Primary breast cancer	2062	N	N	Pretreatment	I–IV	30	DFS, OS	5	DFS, OS	Yes	8
Nakamura	2017	Japan	Asia	Primary breast cancer	208	93.6	55.3	Not clear	0–IV	27	OS	5	OS	No	7
Nan	2017	China	Asia	Primary breast cancer	46	27	60	Pretreatment	Not clear	N	N	2.885	OS	No	5
Nieder	2015	Norway	Europe	Breast cancer with brain metastases	14	13.8		Before recurrence	M	77	OS	N	N	No	5
Nishimiya	2014	Japan	Asia	Primary breast cancer	247	120	51.7	Pretreatment	I, II, III	28	DFS, OS	2.5	DFS, OS	No	8
Nisman	2013	Israel	Asia	Primary early breast cancer	159	76	N	Pretreatment	I, II, III	30.0	DFS	N	N	Yes	6
Park	2008	Korea	Asia	Early-stage breast cancer	740	37.2	47	Pretreatment	I, II, III	20.11	DFS, OS	3.88	DFS, OS	Yes	8
Pierga	2012	France	Europe	Metastatic breast cancers	84	14.9	57	After recurrence	M	N	N	N	PFS	No	6
Rasmy	2016	Egypt	Africa	Early-stage breast cancer	280	33.18	49	Pretreatment	I, II, III	25	DFS, OS	N	N	Yes	7
Richard	1988	USA	America	Stage II and III breast cancer	529	72	N	Pretreatment	II, III	N	N	20	DFS, OS	Yes	8
Shao	2015	China	Asia	Early-stage breast cancer	432	N	50	Pretreatment	I, II, III	25	DFS, OS	5.0	DFS, OS	Yes	8
Shering	1998	Ireland	Europe	Operable breast cancer	368	39.36	56	Pretreatment	I, II, III	30	DFS, OS	N	N	No	7
Turanli	2010	Turkey	Europe	Isolated bone metastasis breast cancer	129	38	51	After bone metastasis	M	30	PFS, OS	N	N	Yes	7
Uehara	2008	Japan	Asia	Primary breast cancer	1663	N	N	Pretreatment	I–IV	28	DFS, OS	5.0	DFS, OS	No	7
Velaiutham	2008	Malaysia	Asia	Primary breast cancer	322	N	N	Pretreatment	I–IV	50	OS	N	N	No	5
Wang	2014	China	Asia	Breast cancer	86	N	N	Not clear	Not clear	25.00	OS	3.40	OS	No	5
Wu	2014	China	Asia	Early-stage breast cancer	470	49.9	48	Pretreatment	I, II, III	25	DFS, OS	5.0	DFS, OS	Yes	7
Zhao	2011	China	Asia	Stage IV breast cancer with bone metastases	60	31.4	48.5	Pretreatment	M	25	PFS	10	PFS	No	8

OS: overall survival; PFS: progress-free survival; DFS: disease-free survival; M: metastasis; N: not available; NOS: Newcastle–Ottawa Scale.

**Table 2:** Summary of the meta-analysis results.

Analysis	Univariate analysis					Multivariate analysis				
	*N*	HR (95% CI)	*p*	*I* ^2^	Ph	*N*	HR (95% CI)	*p*	*I* ^2^	Ph
CA15-3 DFS										
All	14	2.61 (2.17–3.13)	<0.001	50	0.02	10	1.56 (1.38–1.76)	<0.001	31	0.16
Subgroup 1										
Primary early-stage breast cancer	9	2.57 (2.14–3.08)	<0.001	21	0.25	7	1.80 (1.53–2.11)	<0.001	0	0.63
Metastatic breast cancer	2	2.00 (1.46–2.74)	<0.001	0	0.69	1	1.28 (0.43–3.80)	0.65	—	—
Primary all-stage or unclear-stage breast cancer	3	2.88 (1.54–5.41)	<0.001	82	0.004	2	1.28 (1.06–1.55)	<0.001	40	0.20
Subgroup 2										
Cut-off ≤ 25	6	2.84 (2.30–3.50)	<0.001	0	0.77	5	1.80 (1.50–2.16)	<0.001	0	0.85
Cut-off > 25	6	2.69 (1.87–3.87)	<0.001	75	0.001	4	1.39 (1.17–1.64)	<0.001	59	0.06
Subgroup 3										
Asia and Africa	8	3.18 (2.46–4.11)	<0.001	44	0.08	6	1.96 (1.57–2.44)	<0.001	0	0.65
Europe and America	6	2.14 (1.82–2.52)	<0.001	0	0.67	4	1.41 (1.22–1.63)	<0.001	24	0.27
Subgroup 4										
Sample size < 500	11	2.37 (2.00–2.81)	<0.001	20	0.26	7	1.78 (1.50–2.10)	<0.001	0	0.65
Sample size ≥ 500	3	3.36 (2.01–5.61)	<0.001	79	0.008	3	1.35 (1.13–1.61)	0.001	50	0.14
Subgroup 5										
Publish year > 2010	7	2.82 (2.3–3.48)	<0.001	0	0.45	6	1.80 (1.50–2.17)	<0.001	0	0.53
Publish year ≤ 2010	7	2.45 (1.86–3.23)	<0.001	69	0.004	4	1.39 (1.19–1.64)	<0.001	39	0.18
CA15-3 OS										
All	21	2.86 (2.31–3.54)	<0.001	72	<0.0001	15	2.03 (1.76–2.33)	<0.001	0	0.45
Subgroup 1										
Primary early-stage breast cancer	7	2.95 (2.28–3.82)	<0.001	22	0.26	6	1.93 (1.46–2.55)	<0.001	0	0.84
Metastatic breast cancer	6	1.79 (1.51–2.12)	<0.001	0	0.66	3	1.85 (1.47–2.32)	<0.001	0	0.78
Primary all-stage or unclear-stage breast cancer	8	3.87 (2.74–5.46)	<0.001	74	<0.0001	6	2.31 (1.83–2.93)	<0.001	47	0.09
Subgroup 2										
Cut-off ≤ 25	5	3.11 (2.39–4.05)	<0.001	0	0.59	5	1.98 (1.46–2.69)	<0.001	0	0.90
Cut-off > 25	10	3.57 (2.62–4.88)	<0.001	74	<0.0001	7	2.24 (1.79–2.80)	<0.001	46	0.09
Subgroup 3										
Asia and Africa	11	3.50 (2.59–4.71)	<0.001	70	0.0002	8	1.93 (1.58–2.35)	<0.001	34	0.16
Europe and America	10	2.35 (1.74–3.18)	<0.001	72	0.0002	7	2.13 (1.74–2.60)	<0.001	0	0.81
Subgroup 4										
Sample size < 500	18	2.87 (2.24–3.7)	<0.001	75	<0.0001	12	2.03 (1.74–2.38)	<0.001	21	0.23
Sample size ≥ 500	3	2.72 (1.96–3.78)	<0.001	37	0.20	3	1.99 (1.43–2.77)	<0.001	0	0.99
Subgroup 5										
Publish year > 2010	10	3.17 (2.33–4.31)	<0.001	62	0.005	8	1.82 (1.52–2.18)	<0.001	0	0.90
Publish year ≤ 2010	11	2.65 (1.94–3.62)	<0.001	79	<0.0001	7	2.41 (1.92–3.03)	<0.001	21	0.27
CEA DFS										
All	10	2.60 (2.23–3.04)	<0.001	0	0.91	9	1.77 (1.53–2.04)	<0.001	0	0.82
Subgroup 1										
Primary early-stage breast cancer	6	2.80 (2.24–3.52)	<0.001	0	0.48	5	1.94 (1.56–2.40)	<0.001	0	0.72
Metastatic breast cancer	1	2.31 (1.26–4.24)	0.007	—	—	2	1.91 (1.22–3.00)	0.005	0	0.71
Primary all-stage or unclear-stage breast cancer	3	2.45 (1.94–3.09)	<0.001	0	0.80	2	1.58 (1.28–1.96)	<0.001	0	0.56
Subgroup 2										
Cut-off ≤ 5	6	2.77 (2.29–3.37)	<0.001	0	0.48	6	1.74 (1.49–2.04)	<0.001	0	0.54
Cut-off > 5	3	2.31 (1.72–3.10)	<0.001	0	0.98	1	1.84 (1.07–3.16)	<0.001	—	—
Subgroup 3										
Asia and Africa	6	2.65 (2.19–3.20)	<0.001	0	0.46	5	1.95 (1.53–2.48)	<0.001	0	0.89
Europe and America	4	2.50 (1.89–3.32)	<0.001	0	0.80	4	1.67 (1.40–2.00)	<0.001	0	0.52
Subgroup 4										
Sample size < 500	6	2.62 (2.11–3.24)	<0.001	0	0.54	7	1.87 (1.54–2.26)	<0.001	0	0.97
Sample size ≥ 500	4	2.59 (2.06–3.25)	<0.001	0	0.64	2	1.65 (1.33–2.05)	<0.001	59	0.12
Subgroup 5										
Publish year > 2010	5	2.79 (2.15–3.60)	<0.001	0	0.50	7	1.87 (1.54–2.26)	<0.001	0	0.97
Publish year ≤ 2010	5	2.50 (2.05–3.04)	<0.001	0	0.74	2	1.65 (1.33–2.05)	<0.001	59	0.12
CEA OS										
All	16	2.46 (1.93–3.15)	<0.001	70	<0.0001	8	1.72 (1.49–1.99)	<0.001	0	0.69
Subgroup 1										
Primary early-stage breast cancer	7	3.68 (2.65–5.09)	<0.001	33	0.17	4	1.95 (1.49–2.57)	<0.001	0	0.89
Metastatic breast cancer	4	1.52 (1.27–1.82)	<0.001	0	0.41	1	1.37 (1.02–1.85)	0.04	—	—
Primary all-stage or unclear-stage breast cancer	5	2.61 (1.88–3.62)	<0.001	42	0.14	3	1.79 (1.46–2.20)	<0.001	0	0.64
Subgroup 2										
Cut-off ≤ 5	8	3.80 (2.67–5.39)	<0.001	59	0.02	6	1.86 (1.56–2.20)	<0.001	0	0.88
Cut-off > 5	4	2.34 (1.73–3.17)	<0.001	0	0.96	1	1.77 (1.03–3.03)	<0.001	—	—
Subgroup 3										
Asia and Africa	11	2.82 (2.07–3.85)	<0.001	72	<0.001	7	1.65 (1.38–1.96)	<0.001	0	0.70
Europe and America	5	1.87 (1.26–2.77)	0.002	61	0.04	1	1.91 (1.48–2.46)	<0.001	—	—
Subgroup 4										
Sample size < 500	13	2.48 (1.84–3.34)	<0.001	75	<0.0001	6	1.64 (1.37–1.95)	<0.001	0	0.60
Sample size ≥ 500	3	2.29 (1.75–3.00)	<0.001	0	0.48	2	1.91 (1.50–2.45)	<0.001	0	0.96
Subgroup 5										
Publish year > 2010	9	3.27 (2.22–4.82)	<0.001	73	<0.0001	6	1.64 (1.37–1.95)	<0.001	0	0.60
Publish year ≤ 2010	7	1.82 (1.39–2.39)	<0.001	53	0.05	2	1.91 (1.50–2.45)	<0.001	0	0.96

N: number of studies; HR: hazard ratio; 95% CI: 95% confidence interval; Ph: *p* values of *Q* test for heterogeneity test; OS: overall survival; DFS: disease-free survival; “—” means unavailable.

**Table 3:** Meta-analysis of the association between CEA or CA15-3 and clinicopathological features of breast cancer.

Variables	CEA							CA15-3						
	Number of studies	Number of patients	OR (95% CI)	*P*	Heterogeneity *I* ^2^ (%)	Heterogeneity Ph	Mode	Number of studies	Number of patients	OR (95% CI)	*P*	Heterogeneity *I* ^2^ (%)	Heterogeneity Ph	Mode
Age (≤35 versus >35)	3	1210	0.52 (0.29–0.94)	0.03	10	0.33	Fixed	3	1251	1.83 (1.20–2.80)	0.005	0	0.87	Fixed
Menopause (no versus yes)	3	792	0.75 (0.33–1.69)	0.49	62	0.07	Random	5	1524	1.16 (0.90–1.50)	0.25	19	0.29	Fixed
Tumor size (≤5 cm versus >5 cm)	5	1385	0.28 (0.13–0.57)	<0.001	69	0.01	Random	9	2766	0.42 (0.29–0.63)	<0.001	62	0.007	Random
Lymph node metastasis (no versus yes)	6	3497	0.44 (0.36–0.53)	<0.001	13	0.33	Fixed	9	4867	0.59 (0.38–0.93)	0.02	84	<0.001	Random
TNM stage (I-II versus III-IV)	7	4706	0.39 (0.25–0.60)	<0.001	77	0.0002	Random	10	5298	0.34 (0.20–0.58)	<0.001	88	<0.001	Random
Histological grade (I-II versus III)	5	2693	0.87 (0.58–1.31)	0.50	53	0.07	Random	8	3980	0.63 (0.46–0.87)	0.005	64	0.006	Random
ER (no versus yes)	5	3047	0.75 (0.41–1.38)	0.36	81	0.0003	Random	8	4194	1.23 (0.93–1.61)	0.14	50	0.05	Random
PR (no versus yes)	3	2622	0.83 (0.47–1.46)	0.52	78	0.01	Random	7	3810	1.01 (0.86–1.18)	0.95	0	0.91	Fixed
HER-2 (no versus yes)	2	730	0.67 (0.25–1.78)	0.42	83	0.01	Random	5	1305	1.11 (0.83–1.47)	0.48	0	0.76	Fixed
Luminal type (no versus yes)	3	1200	0.72 (0.37–1.39)	0.33	63	0.07	Random	4	1396	1.32 (0.77–2.22)	0.31	65	0.04	Random
HER-2 overexpress type (no versus yes)	3	1200	0.78 (0.32–1.89)	0.57	67	0.05	Random	4	1396	0.75 (0.50–1.14)	0.18	46	0.13	Fixed
Triple-negative type (no versus yes)	3	1200	2.08 (1.30–3.33)	0.003	52	0.12	Fixed	4	1396	0.91 (0.65–1.28)	0.59	0	0.51	Fixed

OR: odds ratio; 95% CI: 95% confidence interval; Ph: *p* values of *Q* for heterogeneity test; ER: estrogen receptor; PR: progesterone receptor; HER-2: human epidermal growth factor receptor 2.

## Additional Points

*Writing Assistance*. This article received English editing from American Journal Experts (certificate verification key: E6F4-11E9-CF02-D4A3-CEF3).

## Supplementary Materials

Supplementary Materials Figure 1: Begg's funnel plot of the study: (a) univariate result of DFS of CA15-3; (b) multivariate result of DFS of CA15-3; (c) univariate result of OS of CA15-3; (d) multivariate result of OS of CA15-3; (e) univariate result of DFS of CEA; (f) multivariate result of DFS of CEA; (g) univariate result of OS of CEA; (h) multivariate result of OS of CEA.
