# Supplementary figures and images for: Clinicopathological and Prognostic Significance of Cancer Antigen 15-3 and Carcinoembryonic Antigen in Breast Cancer: A Meta-Analysis including 12,993 Patients
Source: Dis Markers. 2018 May 2;2018:9863092. doi: 10.1155/2018/9863092 (PMC5954898; doi:10.1155/2018/9863092)

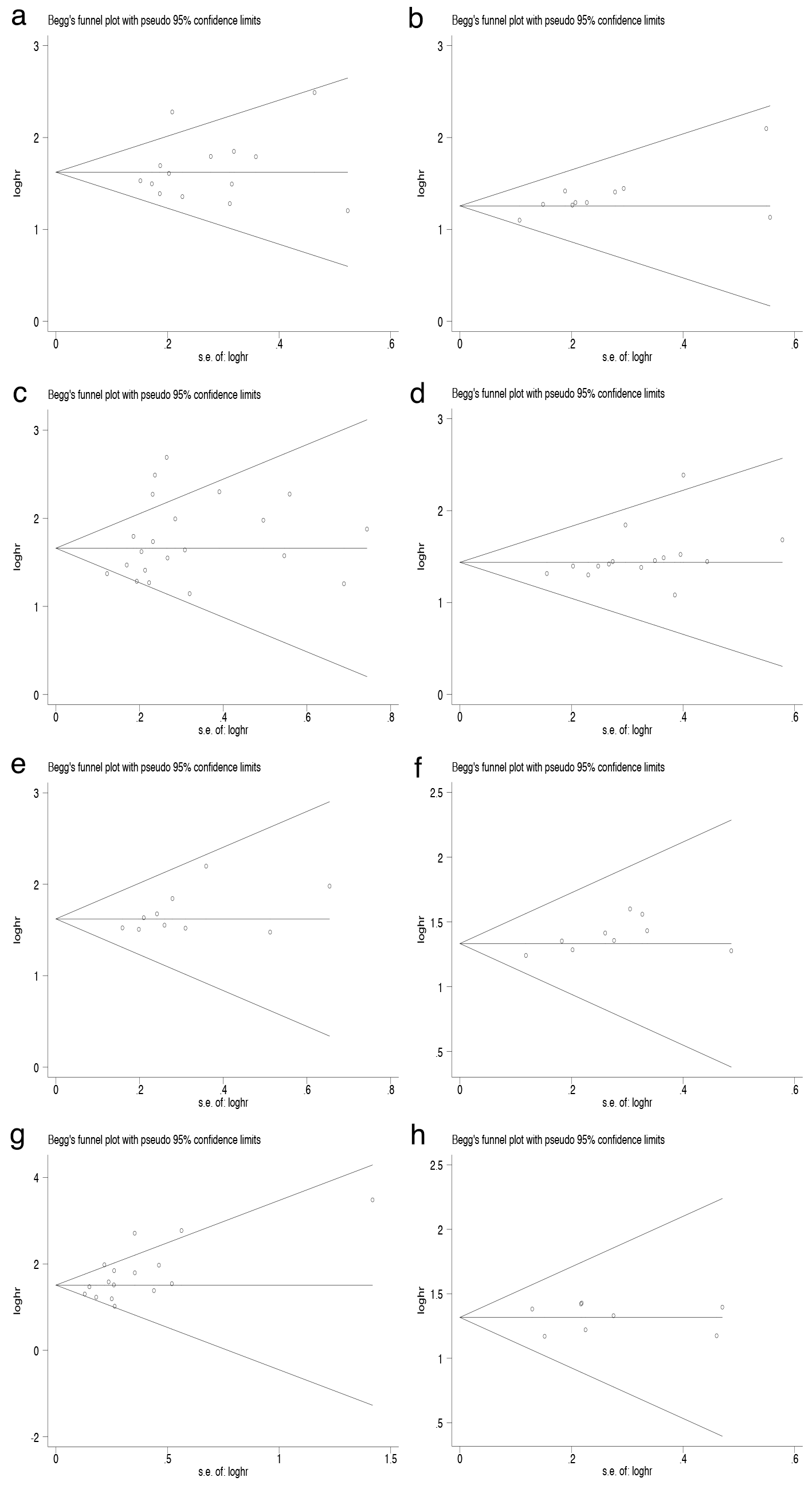

Supplement: Supplementary Materials — Figure 1: Begg's funnel plot of the study: (a) univariate result of DFS of CA15-3; (b) multivariate result of DFS of CA15-3; (c) univariate result of OS of CA15-3; (d) multivariate result of OS of CA15-3; (e) univariate result of DFS of CEA; (f) multivariate result of DFS of CEA; (g) univariate result of OS of CEA; (h) multivariate result of OS of CEA. [file 9863092.f1.doc]
